# Current Pharmacotherapeutic Strategies in Diffuse Gliomas: Focus on Glioblastoma, IDH-Wildtype, and Emerging Targeted Therapies for IDH-Mutant Tumors

**DOI:** 10.3390/ph19010148

**Published:** 2026-01-14

**Authors:** Klaudia Dynarowicz, Barbara Smolak, Dorota Bartusik-Aebisher, Wiesław Guz, Gabriela Henrykowska, David Aebisher

**Affiliations:** 1 Department of Biochemistry and General Chemistry, Faculty of Medicine, University of Rzeszów, 35-310 Rzeszów, Poland; kdynarowicz@ur.edu.pl (K.D.); dbartusikaebisher@ur.edu.pl (D.B.-A.); 2 Department of Diagnostic Imaging and Nuclear Medicine, Faculty of Medicine, University of Rzeszów, 35-310 Rzeszów, Poland; bsmolak@ur.edu.pl; 3 Department of Epidemiology and Public Health, Faculty of Medicine, Medical University of Lodz, 90-419 Lodz, Poland; gabriela.henrykowska@umed.lodz.pl; 4 Department of Photomedicine and Physical Chemistry, Faculty of Medicine, University of Rzeszów, 35-310 Rzeszów, Poland

**Keywords:** glioblastoma, IDH-wildtype, astrocytoma, IDH-mutant, drug candidate, brain tumor, ivosidenib

## Abstract

Glioblastoma, isocitrate dehydrogenase (*IDH1/2*) wild-type (IDH-wildtype), is one of the most aggressive and malignant tumors of the central nervous system, characterized by rapid growth, pronounced cellular heterogeneity, and an exceptionally poor prognosis. The median survival time for patients with glioblastoma, IDH-wildtype, is approximately 15 months after diagnosis, and current multimodal treatment strategies remain largely ineffective. This review focuses on contemporary pharmacotherapeutic approaches used in the management of glioblastoma, IDH-wildtype, including temozolomide-based chemotherapy, corticosteroids for edema control, and antiangiogenic therapy in recurrent disease, with particular emphasis on their clinical efficacy and limitations. In addition, the review discusses emerging targeted therapeutic strategies developed for IDH-mutant diffuse gliomas, which represent a biologically distinct disease entity. Particular attention is given to ivosidenib, a selective inhibitor of mutant *IDH1*, currently evaluated for the treatment of astrocytoma, IDH-mutant, grade 4. Its epigenetic mechanism of action, involving inhibition of the oncometabolite 2-hydroxyglutarate (2-HG), is outlined, along with preliminary clinical evidence suggesting potential to delay disease progression. Finally, innovative drug-delivery technologies designed to overcome the blood–brain barrier are briefly discussed as complementary strategies that may enhance the efficacy of both conventional and targeted therapies. Overall, future advances in the treatment of diffuse gliomas will likely depend on the integration of molecularly targeted agents, predictive biomarkers, and advanced delivery platforms aimed at improving patient survival and quality of life.

## 1. Introduction

Glioblastoma, IDH-wildtype, is the most aggressive primary malignant tumor of the central nervous system in adults and remains associated with an exceptionally poor prognosis. Despite advances in neurosurgical techniques, radiotherapy, chemotherapy, and neuroimaging, median survival rarely exceeds 15 months from diagnosis, and long-term survival remains uncommon [1,2]. A defining biological feature of glioblastoma is its highly infiltrative growth pattern, which precludes complete surgical resection without unacceptable neurological risk due to invasion of functionally critical brain regions [3].

According to the 2021 World Health Organization (WHO) classification of tumors of the central nervous system, the term glioblastoma is now restricted exclusively to diffuse astrocytic tumors of grade 4 that lack mutations in the isocitrate dehydrogenase (*IDH*) genes. Diffuse gliomas harboring *IDH1* or *IDH2* mutations are no longer classified as glioblastoma and are instead designated as astrocytoma, IDH-mutant, grade 4, reflecting their distinct molecular background and more favorable clinical course [1,2]. This reclassification has important diagnostic and therapeutic implications and necessitates a clear distinction between treatment strategies developed for glioblastoma, IDH-wildtype, and those intended for IDH-mutant diffuse gliomas.

Standard treatment for glioblastoma, IDH-wildtype, is based on a multimodal approach consisting of maximally safe surgical resection followed by radiotherapy and concomitant and adjuvant chemotherapy with temozolomide (TMZ), an oral alkylating agent [4,5]. While this regimen remains the cornerstone of first-line therapy, its clinical effectiveness is limited by the frequent development of treatment resistance. Although the activity of the DNA repair enzyme O^6^-methylguanine-DNA methyltransferase (MGMT) is known to influence tumor sensitivity to TMZ, the molecular mechanisms underlying resistance are incompletely understood and likely involve multiple adaptive processes [6,7]. An additional therapeutic challenge is posed by the blood–brain barrier, which restricts drug penetration and reduces intratumoral drug concentrations [8].

Glioma stem-like cells (GSCs) have been implicated in tumor recurrence and therapeutic resistance in glioblastoma. These cells exhibit enhanced capacity for self-renewal and survival under therapeutic stress and have been shown to regenerate heterogeneous tumor populations in experimental models [9]. However, direct in vivo evidence demonstrating that a single GSC can initiate tumor regrowth in patients is currently lacking, underscoring the need for cautious interpretation of experimental findings [10]. In parallel, glioblastoma is characterized by a profoundly immunosuppressive tumor microenvironment that impairs antitumor immune responses through cytokine secretion and expression of immune checkpoint molecules such as PD-L1 [11,12].

In response to these challenges, significant efforts have been devoted to the development of novel therapeutic strategies for glioblastoma, IDH-wildtype, including immunotherapy, cancer vaccines, and targeted radionuclide approaches [13,14,15,16,17,18]. Advances in molecular imaging and biomarker-driven patient stratification further support the concept of personalized treatment strategies [19,20]. Nevertheless, the clinical benefit of many of these approaches remains limited, highlighting the urgent need for more effective and durable therapies.

**Figure 1:**
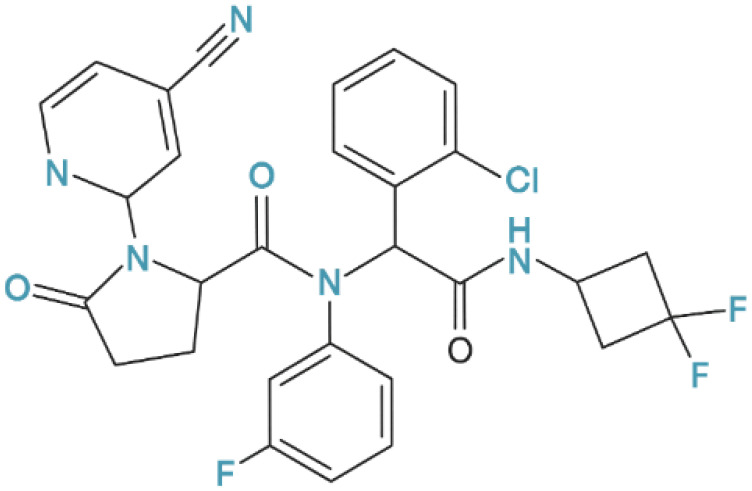
Chemical structure of the selective mutant IDH1 inhibitor AG-120 (ivosidenib).

At the same time, growing interest has emerged in molecularly targeted therapies developed for IDH-mutant diffuse gliomas, which represent a biologically distinct disease entity from glioblastoma, IDH-wildtype. Inhibitors of mutant *IDH1* constitute a rational therapeutic strategy for these tumors by targeting aberrant production of the oncometabolite 2-hydroxyglutarate (2-HG). Among these agents, ivosidenib (AG-120) has attracted considerable attention as a selective small-molecule inhibitor of mutant *IDH1*. Importantly, IDH inhibitors are not intended for the treatment of glioblastoma, IDH-wildtype, but are discussed in this review in the context of IDH-mutant diffuse gliomas. The chemical structure of ivosidenib is presented in Figure 1, and its pharmacological properties and emerging clinical evidence are discussed in a dedicated section.

## 2. Contemporary Approaches to Glioblastoma: Epidemiology, Pharmacotherapy, and Emerging Drug-Delivery Technologies

### 2.1. Epidemiology and Significance of the Problem

In this section, the term glioblastoma refers exclusively to glioblastoma, IDH-wildtype, in accordance with the WHO 2021 classification.

Glioblastoma, IDH-wildtype, is the most common primary malignant tumor of the central nervous system in adults, accounting for approximately 15–20% of all intracranial tumors and nearly half of all glial malignancies [21,22]. It is characterized by aggressive growth, limited therapeutic options, and persistently poor clinical outcomes, making it a major global health challenge. The incidence of glioblastoma is estimated at approximately 3–5 cases per 100,000 individuals per year, with relatively stable rates observed across developed countries [2].

Epidemiological trends indicate that glioblastoma predominantly affects older adults, with a median age at diagnosis of approximately 65 years, and occurs more frequently in men than in women, with a male-to-female ratio of approximately 1.6:1 [2,23]. While glioblastoma can occur in younger individuals, including pediatric patients, such cases account for less than 3% of all diagnoses [22]. Diffuse gliomas harboring IDH mutations are more commonly diagnosed in younger patients and are associated with a more favorable prognosis, highlighting their biological distinction from glioblastoma, IDH-wildtype.

The observed increase in the number of glioblastoma diagnoses over recent decades is largely attributed to population aging and improved access to advanced neuroimaging techniques, such as MRI and CT, rather than a true rise in disease incidence [24,25]. Differences in reported incidence among ethnic groups have been described; however, these variations likely reflect a combination of genetic background, environmental factors, and disparities in healthcare access.

In summary, glioblastoma remains the most aggressive primary brain tumor in adults, with a high but relatively stable incidence that correlates strongly with age and sex. Despite advances in diagnostics, its epidemiological profile underscores the urgent need for more effective therapeutic strategies rather than further descriptive population analyses.

#### 2.1.1. Risk Factors and Predispositions

The etiology of glioblastoma, IDH-wildtype, remains incompletely understood. Most cases arise sporadically, with no single environmental or hereditary factor sufficient to explain disease development [26]. Current evidence suggests that glioblastoma results from a complex interplay between genetic susceptibility, environmental exposure, and epigenetic dysregulation.

Exposure to ionizing radiation remains the only well-established environmental risk factor for glioblastoma. Individuals who received cranial radiotherapy during childhood—for example, as part of treatment for acute lymphoblastic leukemia—demonstrate a significantly increased risk of developing secondary high-grade gliomas later in life [26,27]. This association is attributed to radiation-induced DNA damage in neural precursor cells.

Other environmental factors, including electromagnetic field exposure, pesticide contact, and tobacco smoking, have been investigated; however, large epidemiological studies have failed to demonstrate a consistent causal relationship between these exposures and glioblastoma development [28]. These factors, if relevant, are thought to exert only a minor modulatory effect in genetically predisposed individuals.

Although inherited genetic syndromes account for only a small proportion of cases, they provide important insights into glioblastoma pathogenesis. Hereditary cancer predisposition syndromes such as Li-Fraumeni and Turcot syndrome are associated with an increased risk of glioma development, reflecting the role of impaired DNA damage response and genomic instability in tumor initiation [29,30].

Mutations in *IDH1* and *IDH2* genes, while not risk factors for glioblastoma, IDH-wildtype, are characteristic of IDH-mutant diffuse gliomas and serve as critical diagnostic and prognostic biomarkers. Their presence defines a biologically distinct disease entity with a more favorable clinical course, underscoring the importance of molecular classification in modern neuro-oncology [30].

Additional research has explored the contribution of polymorphisms in DNA repair genes (e.g., ERCC1, XRCC1) and epigenetic alterations, such as aberrant DNA methylation, to glioblastoma susceptibility. However, current evidence remains insufficient to establish these factors as independent risk determinants, and their clinical relevance requires validation in larger population-based studies [31,32].

In summary, while ionizing radiation remains the only confirmed risk factor for glioblastoma, increasing evidence supports the contribution of genetic and epigenetic susceptibility in disease development. Understanding these interactions is essential for future efforts in risk stratification and the development of personalized therapeutic strategies rather than population-level prevention.

#### 2.1.2. Clinical and Social Significance

Glioblastoma, IDH-wildtype, represents one of the greatest challenges in modern neuro-oncology due to its aggressive biological behavior, high invasiveness, and extremely limited therapeutic options. Despite advances in diagnostic imaging, microsurgical techniques, and multimodal treatment protocols, clinical outcomes remain poor, with median survival rarely exceeding 12–18 months [33,34]. This unfavorable prognosis is largely attributable to the profound molecular heterogeneity of the tumor, which promotes rapid development of therapeutic resistance and inevitable disease recurrence [35].

The current standard of care, known as the Stupp regimen, combines maximal safe surgical resection with radiotherapy and concomitant and adjuvant TMZ chemotherapy [33]. Even with modern surgical adjuncts such as neuronavigation, intraoperative MRI, and 5-aminolevulinic acid (5-ALA) fluorescence, complete tumor resection is virtually impossible due to the infiltrative growth pattern of glioblastoma leading to frequent local relapse [35]. An additional major limitation to treatment efficacy is the blood–brain barrier, which restricts the penetration of both cytotoxic and targeted therapeutic agents into tumor tissue [35,36].

In recent years, novel therapeutic approaches—including immunotherapy, molecularly targeted agents, oncolytic viruses, gene therapy, and Tumor Treating Fields (TTFields)—have been explored in an effort to improve outcomes in selected patient populations [37]. Although some of these strategies have demonstrated modest survival benefits, their broader clinical application remains limited by cost, accessibility, and variable efficacy.

Beyond its medical implications, glioblastoma imposes a substantial social and economic burden. Neurological deficits, cognitive impairment, and progressive loss of independence significantly reduce patient quality of life and generate high demands on healthcare systems and caregivers [36,38]. From a public health perspective, the combination of high morbidity and poor survival results in a significant loss of quality-adjusted life years (QALYs), underscoring the urgent need for more effective therapeutic strategies [39].

#### 2.1.3. Global Epidemiological Data

The global incidence of glioblastoma, IDH-wildtype, exhibits regional variation, influenced by demographic structure, genetic background, and differences in diagnostic availability and cancer registration quality. In North America and Western Europe, incidence rates range from approximately 3 to 4 cases per 100,000 individuals per year, making glioblastoma the most common primary malignant tumor of the central nervous system in adults (Table 1) [40,41]. In contrast, lower reported incidence rates are observed in Asia, South America, and Sub-Saharan Africa, likely reflecting underdiagnosis and limited access to advanced neuroimaging rather than true biological differences [42,43].

According to recent global analyses based on the GLOBOCAN 2022 database, approximately 322,000 new cases of brain and central nervous system tumors are diagnosed annually worldwide, with glioblastoma accounting for the largest proportion of malignant entities [44]. Across regions, glioblastoma predominantly affects older adults, with peak incidence in the sixth and seventh decades of life, and shows a consistent male predominance [22,26,47]. Survival outcomes vary substantially between high- and low-income countries, largely reflecting disparities in access to multidisciplinary care, modern surgical techniques, and adjuvant therapies [45,46].

Overall, these epidemiological patterns emphasize that population aging and healthcare infrastructure, rather than changing environmental exposures, are the primary drivers of observed global variation in glioblastoma incidence and outcomes. Understanding these trends is important for healthcare planning; however, they further highlight that meaningful improvements in patient survival will depend primarily on advances in therapeutic strategies rather than epidemiological interventions.

#### 2.1.4. Symptoms

The clinical presentation of glioblastoma, IDH-wildtype, is heterogeneous and depends on tumor location, size, growth dynamics, and the extent of invasion into surrounding brain structures. Symptoms result primarily from focal neural tissue damage and increased intracranial pressure, often leading to progressive neurological deterioration [48].

Common presenting symptoms include persistent headaches, nausea, vomiting, altered consciousness, focal neurological deficits, and epileptic seizures [49,50,51]. Headaches are frequently reported early in the disease course and typically worsen over time, often accompanied by signs of increased intracranial pressure. Seizures represent the initial manifestation in approximately one-quarter of patients and constitute an important component of supportive management [52,53,54,55].

**Figure 2:**
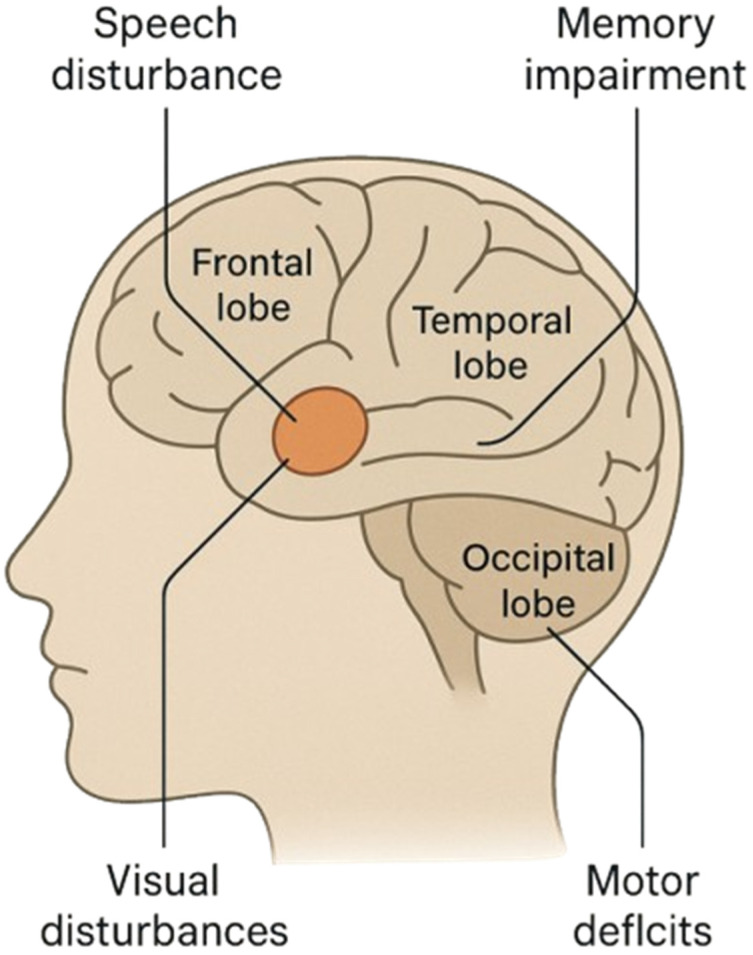
Clinical symptoms and location of glioblastoma. The figure shows the relationship between the tumor’s location within the brain lobes and the predominant neurological symptoms.

As the disease progresses, patients may develop cognitive impairment, personality changes, motor deficits, speech disturbances (Figure 2), and progressive loss of independence, reflecting widespread tumor infiltration and mass effect [52,53,54]. Due to the often nonspecific onset and gradual symptom progression, glioblastoma is frequently diagnosed at an advanced stage, when therapeutic options are limited. Early diagnosis and accurate tumor localization using neuroimaging techniques, particularly magnetic resonance imaging, remain critical for treatment planning and prognostic assessment [56].

#### 2.1.5. Histopathology

Glioblastoma, IDH-wildtype, is a highly aggressive diffuse astrocytic tumor characterized by marked histopathological and molecular heterogeneity, which reflects its invasive behavior and therapeutic resistance [1,57]. This heterogeneity arises from both intrinsic tumor cell diversity and complex interactions with the tumor microenvironment, including endothelial cells, microglia, astrocytes, and extracellular matrix components.

Histopathological diagnosis is based on the evaluation of characteristic microscopic features, including pronounced cellular pleomorphism, high mitotic activity, necrotic areas, and microvascular proliferation. Pseudopalisading necrosis and glomeruloid vascular structures represent hallmark features and constitute key diagnostic criteria according to the WHO classification [57].

The 2021 WHO classification integrates histological and molecular data into a unified diagnostic framework. Within this framework, the diagnosis of glioblastoma is restricted exclusively to IDH-wildtype diffuse astrocytic tumors classified as WHO grade 4, whereas diffuse gliomas harboring *IDH1* or *IDH2* mutations are classified separately as astrocytoma, IDH-mutant, including grade 4. In the absence of classic histological features, the diagnosis of glioblastoma may be established based on specific molecular alterations, including EGFR amplification, TERT promoter mutation, or the combined gain of chromosome 7 and loss of chromosome 10, allowing earlier and more accurate tumor classification [1,57].

Immunohistochemistry plays a crucial role in the integrated diagnosis and prognostication of diffuse gliomas. Markers such as glial fibrillary acidic protein (GFAP) confirm glial origin, while the Ki-67 labeling index provides an estimate of proliferative activity. Importantly, immunohistochemical detection of the *IDH1* R132H mutation constitutes an obligatory diagnostic biomarker in routine clinical practice. Assessment of IDH mutation status is mandatory for the diagnosis of glioblastoma, as the WHO 2021 classification defines this entity exclusively as IDH-wildtype. In cases with negative *IDH1* R132H immunohistochemistry but with histopathological features suggestive of diffuse glioma, additional molecular testing is recommended to exclude non-canonical IDH mutations.

**Figure 3:**
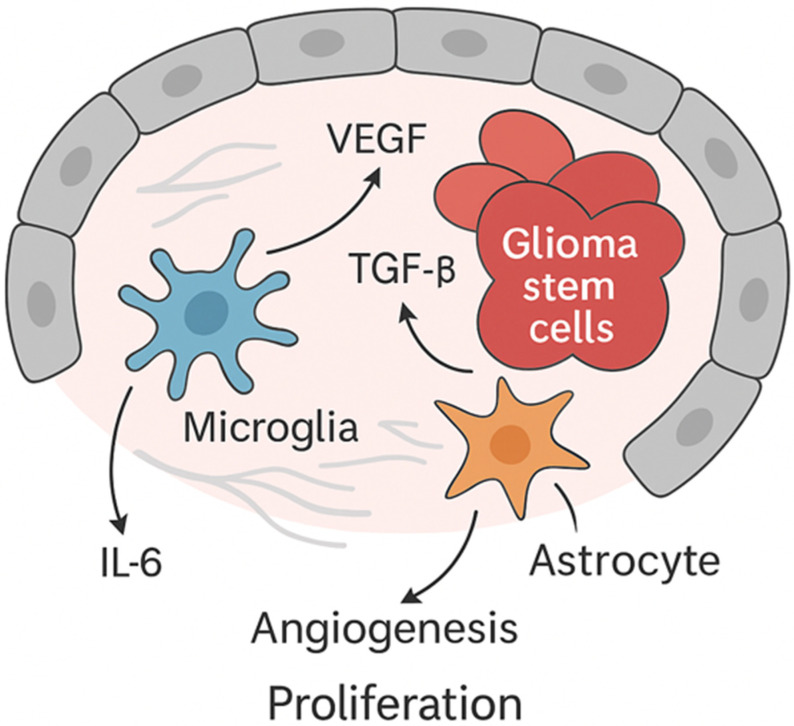
Glioma stem cell niche. The diagram shows the interactions between glioma stem cells, endothelial cells, astrocytes, and microglia in the perivascular niche, indicating the role of VEGF, IL-6, and TGF-β in proliferation and angiogenesis.

Markers associated with glioma stem-like cells, including CD133 and SOX2, are increasingly investigated as research tools reflecting interest in tumor hierarchy and therapeutic resistance; however, they are not currently applied in routine diagnostic practice. Glioma stem-like cells are thought to localize predominantly within perivascular niches, where interactions with endothelial cells and soluble factors such as vascular endothelial growth factor (VEGF), interleukin-6 (IL-6), and transforming growth factor-β (TGF-β) may support tumor maintenance (Figure 3). Nevertheless, their precise clinical relevance and therapeutic implications remain an area of active investigation.

Comprehensive histopathological evaluation, combined with molecular and immunohistochemical analysis, forms the foundation of modern glioblastoma diagnostics and enables improved disease stratification and identification of potential therapeutic targets.

#### 2.1.6. Cell Morphology and Growth Patterns

Glioblastoma, IDH-wildtype, is characterized by marked cellular and nuclear pleomorphism, reflecting profound genetic instability and high proliferative activity [58]. Tumor cells display irregular nuclear morphology, hyperchromasia, prominent nucleoli, and frequent mitotic figures, consistent with dysregulated cell-cycle control [59]. Histologically, the tumor exhibits a heterogeneous architecture with alternating hypercellular and hypocellular regions.

A defining morphological feature of glioblastoma is the presence of necrotic areas surrounded by pseudopalisading tumor cells, which arise as a consequence of regional hypoxia and insufficient vascular perfusion [59]. Microvascular proliferation, including glomeruloid vascular structures, represents another hallmark feature and reflects intense angiogenic signaling within the tumor.

Glioblastoma growth is highly infiltrative, with tumor cells migrating along white matter tracts and perivascular spaces, making complete surgical resection impossible despite advanced intraoperative imaging and navigation techniques [60]. Consequently, microscopic infiltrative foci almost invariably persist after surgery and serve as the basis for tumor recurrence.

Increasing evidence suggests that the aggressive behavior of glioblastoma is not solely determined by its histological appearance but also by aberrant activation of developmental and stemness-related programs. Experimental studies indicate that glioblastoma cells may acquire stem-like properties resembling those of neural progenitor cells; however, the precise cellular origin of glioblastoma remains unresolved and is an area of ongoing investigation [34,61].

#### 2.1.7. Tumor Microenvironment

The tumor microenvironment of glioblastoma, IDH-wildtype, constitutes a complex and dynamic ecosystem that critically influences tumor progression, invasiveness, and therapeutic response [62]. In addition to malignant cells, glioblastoma tissue contains a variety of non-neoplastic components, including astrocytes, endothelial cells, microglia, macrophages, and extracellular matrix (ECM) elements, whose interactions shape tumor behavior.

Microglia and tumor-associated macrophages (TAMs) represent a substantial fraction of the tumor mass and frequently acquire an immunosuppressive phenotype during disease progression. These cells secrete cytokines and growth factors, such as IL-6 and TGF-β, which can promote tumor growth while simultaneously suppressing effective antitumor immune responses, contributing to the immunologically “cold” nature of glioblastoma [62,63,64,65].

Glioma stem-like cells, often detected in perivascular regions, are widely studied as a subpopulation with self-renewal capacity and enhanced survival under therapeutic stress. They are identified experimentally by markers such as CD133 and Nestin and are thought to interact closely with endothelial cells and immune components through paracrine signaling pathways, including VEGF, CXCL12, and Notch [62,63,64,65]. However, their precise contribution to therapy resistance and recurrence in patients has not been conclusively demonstrated in vivo and remains an active area of research.

The extracellular matrix represents a critical structural and signaling component of the glioblastoma microenvironment. Unlike normal adult brain tissue, which is enriched in hyaluronan and chondroitin sulfate proteoglycans, glioblastoma is associated with extensive ECM remodeling. This includes aberrant deposition of fibrillar and basement membrane–associated proteins such as collagens (types I, IV, and VI), laminin, and fibronectin, particularly within perivascular niches and invasive tumor fronts [66,67,68,69]. These ECM components interact with tumor cells via integrin-mediated signaling, promoting cytoskeletal reorganization, migration, and survival. ECM remodeling is further facilitated by increased activity of matrix metalloproteinases (e.g., MMP-2 and MMP-9), enabling infiltrative growth and contributing to therapeutic resistance [66,67,68,69].

Collectively, the glioblastoma microenvironment functions as an active regulator of tumor progression by modulating immune responses, angiogenesis, and cellular plasticity. Targeting microenvironmental components, in addition to tumor cells themselves, represents a promising but still largely experimental therapeutic strategy.

#### 2.1.8. Histopathological and Molecular Classification

Contemporary classification of diffuse gliomas is based on the concept of integrated diagnosis, which combines histopathological features with molecular and genetic alterations, as defined in the 2021 WHO classification [1]. Within this framework, glioblastoma is strictly defined as an IDH-wildtype diffuse astrocytic tumor of grade 4, whereas tumors harboring *IDH1* or *IDH2* mutations are classified separately as astrocytoma, IDH-mutant, grade 4.

Assessment of IDH mutation status represents a cornerstone of modern glioma diagnostics. Immunohistochemical detection of the *IDH1* R132H mutation is routinely performed and constitutes a mandatory biomarker, with additional molecular testing recommended in immunonegative cases with suggestive histological features. This distinction has major prognostic and therapeutic implications, as IDH-mutant tumors exhibit a distinct epigenetic landscape and a more favorable clinical course compared to glioblastoma, IDH-wildtype [70].

Glioblastoma, IDH-wildtype, is characterized by recurrent molecular alterations, including EGFR amplification, TERT promoter mutations, chromosome 7 gain, and chromosome 10 loss, which contribute to aggressive tumor behavior and poor prognosis [71,72]. In contrast, IDH-mutant astrocytomas display widespread DNA hypermethylation (G-CIMP phenotype) driven by accumulation of the oncometabolite 2-hydroxyglutarate, resulting in altered gene expression and impaired cellular differentiation [70].

The methylation status of the MGMT promoter represents an additional clinically relevant biomarker in glioblastoma, IDH-wildtype. MGMT promoter methylation is associated with increased sensitivity to TMZ and improved patient survival, and its assessment is routinely incorporated into clinical decision-making.

#### 2.1.9. Necrotic Areas and Microangiopathy

Areas of necrosis are a hallmark of glioblastoma and result from rapid tumor growth that exceeds vascular supply, leading to chronic hypoxia and accumulation of metabolic byproducts. Necrosis, most commonly coagulative, may occupy a substantial portion of the tumor and is typically surrounded by zones of high proliferative activity. A characteristic histopathological feature is pseudopalisading necrosis, in which tumor cells arrange themselves around central necrotic foci, reflecting directed migration toward regions with improved oxygenation [73].

Vascular abnormalities in glioblastoma manifest as pronounced microangiopathy with endothelial proliferation and formation of glomeruloid vessels. These changes are driven by hypoxia-induced angiogenic signaling, primarily mediated by vascular endothelial growth factor (VEGF), angiopoietin-2, and hypoxia-inducible factor 1α (HIF-1α) [73,74]. Newly formed vessels are structurally abnormal and highly permeable, promoting peritumoral edema and contributing to increased intracranial pressure and neurological deterioration [73].

At the same time, the chaotic and heterogeneous vascular network results in uneven tumor perfusion, which limits effective drug delivery and reduces the efficacy of radiotherapy. These vascular features are considered key contributors to therapeutic resistance and intratumoral heterogeneity in glioblastoma [73,74].

Antiangiogenic therapies, particularly VEGF blockade with agents such as bevacizumab, can transiently normalize tumor vasculature, improve perfusion, and reduce edema, leading to symptomatic relief. However, adaptive tumor responses and activation of alternative angiogenic pathways limit the durability of this effect, preventing sustained survival benefit [74].

#### 2.1.10. Diagnostic Methods and Challenges

Histopathological diagnosis of glioblastoma is a key step in tumor identification and classification and is based on the analysis of tissue samples obtained during stereotactic biopsy or surgical resection. Classical staining techniques, particularly hematoxylin and eosin (H&E), remain the foundation of morphological assessment, enabling evaluation of tumor architecture, cellular atypia, and the presence of hallmark features such as necrosis and microvascular proliferation [75].

Immunohistochemistry constitutes an essential component of glioblastoma diagnostics, providing markers necessary for determining tumor lineage, proliferative activity, and prognostic assessment. GFAP confirms astrocytic origin, while the Ki-67 (MIB-1) proliferation index reflects tumor growth dynamics and correlates with disease aggressiveness, with elevated values commonly observed in glioblastoma [76].

In recent years, diagnostic strategies have been significantly expanded through the incorporation of molecular techniques, including next-generation sequencing, which enables detection of key genetic alterations such as EGFR amplification, chromosomal imbalances, and other clinically relevant molecular events [76,77]. Integration of histopathological findings with molecular profiling forms the basis of the modern concept of integrated diagnosis, consistent with the WHO 2021 classification, and allows for improved tumor stratification and prognostic evaluation [1,77].

One of the major diagnostic challenges in glioblastoma remains intratumoral heterogeneity, which can result in spatially variable morphological and molecular features within a single lesion. This phenomenon necessitates careful tissue sampling and, where feasible, complementary use of advanced imaging modalities, including magnetic resonance imaging and metabolic imaging techniques, to support comprehensive tumor assessment [76,77,78].

#### 2.1.11. Clinical Significance and Future Directions

The histopathology of glioblastoma, IDH-wildtype, plays an important role in prognostic assessment and treatment planning by enabling risk stratification and supporting therapeutic decision-making. Certain microscopic features, including extensive necrotic areas and a high Ki-67 proliferation index, are associated with a more aggressive tumor phenotype and shorter overall survival, which in most patients remains below 15 months. In contrast, diffuse gliomas harboring *IDH1* or *IDH2* mutations—currently classified as astrocytoma, IDH-mutant—are associated with a more favorable clinical course and longer median survival [79]. Integration of histopathological findings with molecular analyses facilitates prediction of response to standard-of-care treatment, including surgical resection followed by radiotherapy and TMZ-based chemotherapy.

Recent advances in molecular diagnostics and multimodal imaging have expanded the possibilities for biologically informed patient stratification and personalized therapeutic approaches. In particular, alterations in signaling pathways such as EGFR and immune checkpoint regulation have provided the rationale for the development of targeted therapies and immunotherapeutic strategies, which are currently under clinical investigation in glioblastoma. In parallel, increasing attention has been directed toward the role of the tumor microenvironment, including microglia, macrophages, and glioma stem-like cells, in shaping tumor progression and modulating treatment response. While these components are widely studied in experimental models, their precise contribution to therapeutic resistance and clinical outcome in patients remains incompletely defined.

From a clinical perspective, further integration of histopathological, molecular, and imaging data is expected to improve diagnostic accuracy and support more individualized treatment strategies. Such integrative approaches provide a conceptual framework for future research aimed at improving therapeutic efficacy in glioblastoma, although their clinical utility requires validation in prospective studies [80,81,82].

### 2.2. Types of Drugs Currently Used for Glioma

Glioblastoma remains one of the most challenging tumors of the central nervous system to treat. Pharmacotherapy constitutes a key component of treatment and is typically combined with surgery, radiotherapy, and supportive care, reflecting the multimodal approach recommended by international guidelines, including those of the European Association for Neuro-Oncology (EANO) and the National Comprehensive Cancer Network (NCCN) [83].

Despite significant advances in diagnosis and treatment, the median survival of patients with glioblastoma, IDH-wildtype, remains limited to approximately 15–18 months after diagnosis, underscoring the urgent need for more effective therapeutic strategies. Pharmacological management encompasses a broad range of agents, including chemotherapeutic drugs, glucocorticosteroids, anticonvulsants, and selected molecularly targeted therapies such as angiogenesis inhibitors and immunotherapeutic agents. Many of these approaches are currently under evaluation in advanced clinical trials aimed at improving survival and quality of life. This chapter provides an overview of drugs currently used in glioblastoma therapy, focusing on their mechanisms of action, clinical efficacy, and limitations, based on available clinical evidence, including randomized controlled trials and meta-analyses [84,85,86].

#### 2.2.1. Chemotherapy

Chemotherapy represents a cornerstone of glioblastoma treatment, particularly when administered in combination with radiotherapy according to the standard Stupp protocol, which involves concomitant chemoradiotherapy followed by adjuvant chemotherapy. This regimen remains the current standard of care and is recommended by both EANO and NCCN guidelines [87].

TMZ is the most widely used chemotherapeutic agent in glioblastoma treatment. It is an oral alkylating agent that induces DNA damage by methylating nucleotides, particularly at the O^6^-methylguanine position, thereby interfering with DNA replication and transcription in tumor cells [88,89].

Clinical studies have demonstrated that the addition of TMZ to radiotherapy results in a modest but statistically significant improvement in overall survival compared with radiotherapy alone, with a median survival benefit of approximately 2–3 months in unselected patient populations. Subsequent studies have suggested that this benefit may be further enhanced in selected patients through combination with additional treatment modalities, such as tumor treating fields [90,91].

The clinical benefit of TMZ therapy is strongly influenced by the methylation status of the MGMT gene promoter. MGMT promoter methylation is associated with increased sensitivity to TMZ and improved clinical outcomes, whereas patients with unmethylated MGMT promoters generally derive less benefit. However, the precise biological mechanisms underlying TMZ resistance remain incompletely understood and may involve multiple processes beyond MGMT activity, including alterations in DNA damage response pathways. Consequently, MGMT promoter methylation status is routinely used as a predictive biomarker to support patient stratification and treatment planning [92].

Other alkylating agents, such as lomustine (CCNU) and carmustine (BCNU), have historically been employed in the treatment of recurrent or refractory glioblastoma. Their clinical use is limited by substantial systemic toxicity, including severe myelosuppression and, in the case of BCNU, dose-dependent pulmonary toxicity. Although these agents exhibit antitumor activity in selected settings, their overall clinical benefit remains modest, and they are generally reserved for specific clinical scenarios, such as disease recurrence or combination regimens, rather than routine first-line therapy [93].

More recent therapeutic strategies include combination chemotherapy regimens, such as lomustine combined with TMZ, which are currently being evaluated in phase III clinical trials. These studies have demonstrated modest improvements in progression-free survival in patients with recurrent glioblastoma, typically on the order of several months, but at the cost of increased treatment-related toxicity, particularly hematological adverse events. In parallel, ongoing research is exploring the potential synergistic effects of TMZ combined with poly(ADP-ribose) polymerase (PARP) inhibitors, as well as novel drug delivery systems, including nanoparticle-based formulations designed to enhance blood–brain barrier penetration and intratumoral drug distribution [94].

#### 2.2.2. Anti-Edematous Drugs

Peritumoral edema is one of the most common and clinically significant complications in glioblastoma wildtype. It results from disruption of the blood–brain barrier (BBB), caused by pathological tumor angiogenesis and increased vascular permeability. These processes promote fluid accumulation within the interstitial space, elevation of intracranial pressure, and worsening of neurological symptoms such as headache, nausea, and motor dysfunction [95].

The primary treatment for tumor-associated edema is the administration of glucocorticosteroids, with dexamethasone remaining the first-line therapeutic option. Standard initial doses typically range from 4 to 16 mg per day, adjusted according to the severity of symptoms, followed by gradual dose reduction to minimize toxicity. Dexamethasone decreases vascular permeability, downregulates VEGF expression, and stabilizes endothelial membranes, which leads to reduction in edema and improvement of neurological status. It also suppresses the production of inflammatory mediators, including prostaglandins and leukotrienes, further contributing to symptomatic relief [96].

Despite its clinical effectiveness, prolonged glucocorticosteroid therapy is associated with significant adverse effects, such as muscle atrophy, hyperglycemia, osteoporosis, increased susceptibility to infections, and general immunosuppression. Evidence also indicates that higher cumulative doses of dexamethasone may negatively influence patient outcomes, particularly in the context of immunotherapy. Therefore, current clinical guidelines recommend the use of the lowest effective doses, typically restricted to the perioperative period or acute symptom exacerbations [97].

As an alternative or adjunct to steroid therapy, VEGF inhibitors such as bevacizumab exhibit anti-edematous properties through normalization of tumor vasculature and reduction in vascular leakage. Clinical trials have demonstrated that bevacizumab can significantly decrease radiographic edema and improve neurological function, although these benefits do not translate into extended overall survival. This therapy is particularly useful in recurrent glioblastoma or in patients who require reduction or discontinuation of steroid treatment. Combined with optimized dosing strategies and improved biomarker-guided monitoring, such methods may contribute to safer and more effective management of peritumoral edema in glioblastoma [97,98].

#### 2.2.3. Anticonvulsants

Seizures occur frequently in patients with glioblastoma and arise from cortical irritation caused by infiltrating tumor cells or peritumoral edema. They represent not only a significant clinical symptom but also a factor that worsens quality of life and complicates the course of oncological treatment. For this reason, the use of antiepileptic drugs (AEDs) constitutes an important component of symptomatic management, aiming to control seizures, stabilize neurological function, and support treatment adherence [99].

Currently, levetiracetam is considered the first-line therapy for both the prevention and treatment of seizures in patients with glioblastoma. It is characterized by a favorable safety profile and a minimal potential for pharmacokinetic interactions with chemotherapeutic agents, including TMZ. Its mechanism of action involves modulation of neurotransmitter release through binding to the synaptic vesicle protein SV2A, leading to effective seizure control in most patients receiving monotherapy [100].

Older antiepileptic drugs, such as phenytoin and carbamazepine, are now used less commonly due to their induction of cytochrome P450 enzymes. This results in accelerated metabolism of chemotherapeutic agents—including TMZ—which may decrease their therapeutic exposure. Additionally, conventional AEDs are more frequently associated with adverse effects such as sedation, cognitive impairment, ataxia, and hepatotoxicity, limiting their applicability in individuals with neurological deficits [101].

An emerging area of interest is the potential anticancer activity of certain AEDs, particularly valproic acid, which inhibits histone deacetylases (HDACs). This mechanism has been associated with pro-apoptotic and antiproliferative effects in glioma cells. Some observational studies suggest that valproic acid may be linked to modest improvements in survival outcomes, making it a candidate for adjunctive therapy in selected patients [102].

Treatment retention defined as sustained effectiveness without the need for medication change tends to be higher with levetiracetam compared with older AEDs, which more often require discontinuation due to adverse effects or drug interactions. Therefore, individualized selection of antiepileptic therapy, considering patient characteristics, seizure type, tolerability, and cancer-treatment interactions, remains a crucial aspect of comprehensive glioblastoma management.

#### 2.2.4. Angiogenesis Inhibitors

Angiogenesis plays a key role in the progression of glioblastoma, enabling rapid tumor growth through the formation of new, abnormal blood vessels with increased permeability and unstable structure. This process is driven by proangiogenic factors, the dominant one being VEGF. Consequently, blocking angiogenic pathways has become one of the most promising therapeutic approaches in glioblastoma treatment, aiming not only to limit tumor growth but also to improve the efficacy of combination therapy [102].

The most studied drug in this group is bevacizumab, a humanized monoclonal antibody directed against VEGF-A. It was approved by the FDA in 2009 for the treatment of recurrent glioblastoma. Its mechanism of action involves neutralizing VEGF, which leads to normalization of abnormal tumor vascularization, reduction in peritumoral edema, and improvement of tissue perfusion and oxygenation. This results in rapid clinical improvement, reduction in tumor volume on imaging studies, and improvement of neurological symptoms. Bevacizumab therapy significantly improves quality of life and reduces symptoms associated with cerebral edema, which is particularly important in patients with recurrent glioblastoma and severe neurological symptoms [103,104].

Bevacizumab’s mechanism of action involves blocking the VEGF–VEGFR signaling pathway, which limits the supply of oxygen and nutrients to tumor cells. However, resistance may develop during treatment, associated with the activation of alternative angiogenic pathways such as FGF (fibroblast growth factor) and PDGF (platelet-derived growth factor) [105]. Therefore, current research is focusing on combination therapies that can counteract these compensatory mechanisms. Combinations of bevacizumab with lomustine have shown improvement in median PFS in patients with recurrent glioblastoma, while new clinical trials are evaluating the efficacy of its combination with checkpoint immunotherapy (e.g., nivolumab) or proton radiotherapy, with promising results in symptom control [106]. Despite proven clinical benefits, therapy with angiogenesis inhibitors is associated with the risk of serious adverse events, such as hypertension, intracranial bleeding, impaired wound healing, and venous thrombosis. Therefore, monitoring of hemodynamic and hematological parameters during therapy is essential. Current research focuses on finding biomarkers predicting treatment response, such as VEGF levels in plasma or the expression of its receptors in tumor tissue, which may enable more effective personalization of antiangiogenic therapy in the future [103,104,105,106].

#### 2.2.5. Immunotherapy and Other Experimental Approaches

Immunotherapy is one of the most dynamically developing research directions in the treatment of glioblastoma, although it is still in the clinical trial phase. The goal of these therapies is to reactivate the body’s immune response against cancer cells by modulating pathways regulating T cell activity and the tumor microenvironment. The most clinically advanced approach is the use of immune checkpoint inhibitors, including nivolumab, a monoclonal antibody that blocks the PD-1 (programmed cell death protein-1) receptor. Blocking the PD-1/PD-L1 interaction reverses tumor-induced immunosuppression, enabling cytotoxic T cell activity against glioblastoma cells. The results of the CheckMate-143 study demonstrated limited efficacy of nivolumab as a single agent (median overall survival—approximately 10 months). However, combinations with other therapeutic modalities, such as bevacizumab or radiotherapy, have shown improved response rates by 10–20% in subgroups of patients with high PD-L1 expression [107,108,109].

Besides checkpoint immunotherapy, research is ongoing on molecularly targeted agents, such as erlotinib, an inhibitor of the epidermal growth factor receptor (EGFR). Despite the theoretical basis for their use, the clinical efficacy of these inhibitors in glioblastoma remains limited, primarily due to the high genetic heterogeneity of the tumor and the presence of resistance mutations [107,108,109].

Another promising avenue of research is gene therapy, which utilizes viral vectors carrying suicide genes or modulating the expression of key oncogenes. Convection-enhanced delivery (CED) methods are being developed simultaneously, allowing for increased drug concentration within the tumor while minimizing systemic toxicity. Phase II trial results indicate promising clinical effects of these methods, but they still require standardization and safety validation. Modern immunotherapeutic strategies, such as CAR-T cell therapy directed against tumor antigens, including EGFRvIII, and peptide vaccines inducing T cell responses to specific tumor epitopes, are particularly promising.

Preliminary results of clinical trials demonstrate an extended time to progression (PFS), particularly in patients with glioblastoma, IDH-wildtype, confirming the potential of these methods for personalized therapy [110,111,112].

#### 2.2.6. Limitations and Future Directions

Resistance to treatment is one of the most serious challenges in glioblastoma therapy and results from its high genetic and molecular heterogeneity and the presence of glioma stem cells. Subpopulations of tumor cells demonstrate the ability to adapt and develop drug escape mechanisms, such as EGFR gene amplification, PTEN gene mutations, or reactivation of the PI3K/AKT and MAPK signaling pathways [113,114].

Another limitation to effective treatment is the blood–brain barrier which significantly limits the bioavailability of most anticancer drugs. Traditional pharmacological molecules often fail to achieve therapeutic concentrations within the tumor, reducing the effectiveness of both chemotherapy and targeted therapies. In response to this challenge, innovative drug delivery strategies are being developed, such as nanoparticles, liposomes, and convection-enhanced delivery systems, which in preclinical models increase drug penetration while minimizing systemic toxicity [115]. Future research focuses on personalizing treatment based on the patient’s molecular profile, using genomic, transcriptomic, and proteomic data to tailor individual therapy. Multidrug strategies combining different mechanisms of action, including immunotherapy with checkpoint inhibitors TTFields, and CAR-T cell therapies, are gaining importance.

In summary, the future of glioblastoma therapy relies on an integrated approach combining bioengineering, immunology, and data analysis, aimed at overcoming resistance, improving drug penetration, and extending patient survival through personalized treatment strategies. Table 2 provides a summary of the medications used to treat glioblastoma to date.

### 2.3. Innovative Drug-Delivery Technologies for Crossing the Blood–Brain Barrier—New Approaches to BBB Penetration—The Use of Cyclodextrins and Heparin Oligosaccharides

One of the major limitations to the effectiveness of pharmacological therapy for glioblastoma remains the BBB, which effectively protects the central nervous system from the entry of most chemical compounds. The BBB, composed of endothelial cells connected by tight junctions and surrounded by pericytes and astrocytic endfeet, plays a key role in maintaining brain homeostasis, yet at the same time restricts the penetration of up to 98% of small hydrophilic drugs. In glioblastoma this problem becomes particularly significant, because despite the increased vascular permeability within the tumor, the transport of therapeutically relevant substances into its core remains insufficient [116].

In recent years, innovative technologies aimed at overcoming this barrier have been intensively developed—encompassing both structural drug modifications (prodrugs) and the design of advanced carrier systems. A particularly promising concept is the use of macromolecular lipid-like carriers based on cyclodextrins, which may enable the transport of hydrophilic drugs across the BBB without the need for chemical modification of the molecule [117].

Cyclodextrins (CDs) constitute a family of cyclic oligosaccharides built from glucopyranose units forming a toroidal structure with a hydrophilic outer surface and a hydrophobic cavity. Thanks to this architecture, they can form inclusion complexes with various chemical compounds, stabilizing them and improving their solubility. β-Cyclodextrin and its derivatives are widely used in pharmacy as drug carriers capable of increasing bioavailability, stability, and controlling the release profile of active substances [118].

In the context of glioblastoma therapy, particular attention has been given to the possibility of modifying cyclodextrins into amphiphilic structures capable of spontaneously forming supramolecular aggregates resembling micelles. Such constructs combine lipophilic properties (facilitating penetration through biological membranes) with hydrophilic domains capable of binding drug molecules [119]. In the proposed model, β-cyclodextrin serves as the core whose hydroxyl groups are selectively functionalized with long alkyl chains and phosphate moieties, giving the molecule an amphiphilic character. In a lipophilic environment, such as the endothelial membrane of brain vessels, these molecules can organize into structures resembling inverted micelles, in which hydrophilic phosphate groups create an internal compartment capable of encapsulating hydrophilic drugs such as model hydrophilic drugs, including L-DOPA, melphalan, baclofen, or gabapentin [120].

**Figure 4:**
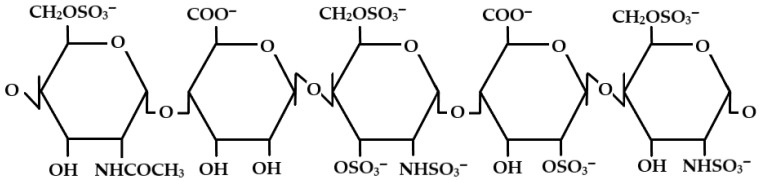
Structure of a heparin fragment (penta-oligosaccharide)—chemical diagram of a glycosaminoglycan capable of crossing the blood–brain barrier [109].

After crossing the BBB and entering the hydrophilic interstitial space of the brain, the macromolecule undergoes reversible reorganization—the phosphate groups become exposed to the outside, leading to destabilization of the complex and controlled release of the active substance within the tumor [121]. This mechanism eliminates the need to modify the chemical structure of the drug (as required for prodrug strategies) and reduces the risk of losing pharmacological activity [122]. Inspiration for this approach comes from observations of heparin oligosaccharides, which can cross the BBB despite their highly anionic nature. In vitro studies have shown that heparin hexa- and octa-oligosaccharides can pass through BBB models in a controlled manner, making them potential design elements for new carrier systems [123]. Heparin (Figure 4), a sulfated glycosaminoglycan, additionally exhibits anti-inflammatory, anti-angiogenic, and anti-metastatic properties that may support glioma therapy by modulating the tumor microenvironment [124].

Integrating heparin motifs into macromolecular cyclodextrin-based carriers may increase their biocompatibility and enable receptor-mediated transport through interactions with endothelial cell-surface proteins. Moreover, these constructs have the potential to induce lipid endocytosis, thereby facilitating uptake into cancer cells via phagocytosis or macropinocytosis [125].

Compared with classical drug-delivery systems—such as liposomes, polymeric nanoparticles, or dendrimers—cyclodextrins offer several advantages: high biodegradability, low immunogenicity, and the ability to precisely control physicochemical properties through chemical modifications. Additionally, due to the enhanced permeability and retention (EPR) effect, macromolecular carriers can accumulate within the tumor, where microvasculature exhibits increased permeability, facilitating selective drug transport [126].

The use of amphiphilic cyclodextrin and heparin derivatives in glioblastoma therapy represents an innovative approach that may significantly improve pharmacotherapy effectiveness by enhancing the bioavailability of active substances, reducing systemic toxicity, and enabling targeted drug release within the tumor. Combining such systems with modern targeted therapies and immunotherapy may form the basis of an integrated glioblastoma—treatment strategy aimed at maximizing therapeutic efficacy while minimizing adverse effects.

### 2.4. Drug Candidate

Glioblastoma is one of the most malignant tumors of the central nervous system, characterized by rapid growth, high invasiveness, and high resistance to treatment. Despite integrated therapeutic management, including surgery, radiotherapy, and chemotherapy, patient prognosis remains poor, with median survival not exceeding 15–18 months [1].

Therapeutic challenges stem from the tumor’s genetic heterogeneity, its ability to invade healthy tissue, and the development of resistance to treatment, leading to almost inevitable disease relapses. Consequently, there is an intensive search for new therapeutic strategies that would increase treatment efficacy while reducing systemic toxicity [1].

Mutations in *IDH1* gene represent one of the most important molecular alterations in diffuse gliomas and constitute a well-defined therapeutic target. *IDH1* mutations occur predominantly in tumors currently classified as astrocytoma, IDH-mutant, including grade 4 according to the WHO 2021 classification, and are associated with profound metabolic and epigenetic reprogramming of tumor cells [127,128]. These alterations provide a strong biological rationale for the development of selective inhibitors targeting mutant *IDH1*, aimed at restoring normal cellular metabolism and differentiation.

One of the most promising research directions is the development of molecularly targeted therapies directed against specific genetic alterations driving gliomagenesis. Of particular importance are *IDH1* mutations, which occur in approximately 10% of diffuse gliomas currently classified as astrocytoma, IDH-mutant, grade 4 according to the WHO 2021 classification. These mutations constitute a well-defined therapeutic target and provide the biological rationale for the development of selective *IDH1* inhibitors such as ivosidenib [129,130].

This mutation leads to the abnormal production of 2-HG, an oncogenic metabolite that disrupts epigenetic processes and cell differentiation. In this context, ivosidenib deserves special attention. It is a selective inhibitor of the mutant IDH1 enzyme, which, by blocking the abnormal catalytic activity, restores normal cellular metabolism and enables tumor cell differentiation. In clinical trials, the drug has demonstrated a favorable safety profile and potential efficacy in the treatment of IDH-mutant diffuse gliomas, making it a candidate for a new drug in targeted therapies with low toxicity [129,130].

#### 2.4.1. Mechanism of Action and Selectivity

Ivosidenib is a highly selective inhibitor of the mutated IDH1 isoenzyme, a key enzyme in the Krebs cycle responsible for the conversion of isocitrate to α-ketoglutarate (α-KG).

Mutant IDH1 enzymes acquire a neomorphic catalytic activity, converting α-ketoglutarate into the oncometabolite D-2-hydroxyglutarate (2-HG), which accumulates at high intracellular concentrations and interferes with α-KG–dependent dioxygenases, including DNA and histone demethylases [127,128]. This leads to widespread epigenetic dysregulation, impaired cellular differentiation, and maintenance of an undifferentiated, tumor-promoting phenotype characteristic of IDH-mutant diffuse gliomas [131].

IDH-mutant glioma cells harboring the R132H mutation in the *IDH1* gene exhibit abnormal changes in enzymatic activity, leading to the accumulation of the oncogenic metabolite 2-hydroxyglutarate. The *IDH1* mutation, most commonly at arginine residue 132 (R132H), occurs primarily in IDH-mutant diffuse gliomas primarily in IDH-mutant diffuse gliomas and results in the conversion of physiological α-KG to 2-HG, which acts as an oncometabolite. Increased 2-HG levels lead to inhibition of α-KG-dependent histone demethylases and DNA demethylases, resulting in global hypermethylation in CpG island regions (so-called G-CIMP, CpG island methylator phenotype). This results in a blockade of progenitor cell differentiation and maintenance of the highly proliferative and invasive cancer phenotype [132,133,134,135].

**Figure 5:**
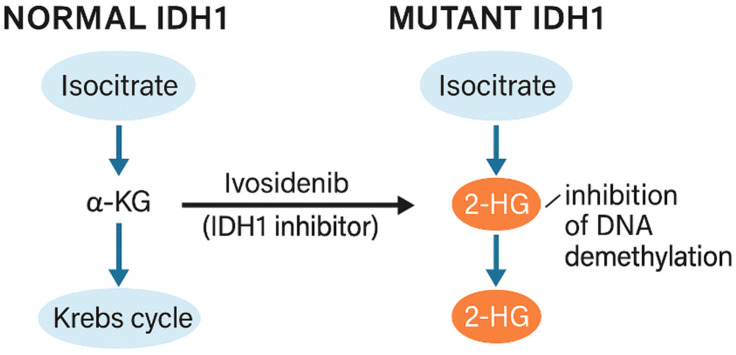
Mechanism of action of ivosidenib in glioma with IDH1 mutation. The diagram shows the differences between normal and mutant forms of IDH1 and the effect of the drug on 2-hydroxyglutarate production and DNA demethylation processes.

Ivosidenib acts by competitively binding to the active site of the mutated IDH1 enzyme, effectively inhibiting 2-HG production and restoring normal epigenetic regulation (Figure 5). Reducing 2-HG levels leads to reactivation of cancer cell differentiation mechanisms, as manifested by increased expression of astrocytic markers such as GFAP and a decreased proliferative phenotype of IDH-mutant glioma cells. Preclinical studies have confirmed that ivosidenib is highly selective towards mutant forms of IDH1, without affecting wild-type isoforms of the enzyme, minimizing the risk of extracellular toxicity and side effects typical of classical chemotherapeutic agents such as TMZ. This makes the drug an example of precision therapy with a favorable safety profile, targeting a specific molecular disorder driving the carcinogenesis process [136,137].

#### 2.4.2. Potential Benefits

Preliminary clinical data indicate that ivosidenib, a selective IDH1 inhibitor, may provide clinical benefit in patients with IDH-mutant diffuse gliomas harboring an *IDH1* mutation—a subgroup in which the effectiveness of standard Stupp-based therapy remains limited. Early-phase clinical trials have demonstrated that ivosidenib effectively reduces intratumoral levels of 2-HG, a hallmark oncometabolite produced by mutant IDH1. This biochemical change correlates with improved radiological findings on MRI, suggesting a slowdown in tumor progression. Reduction of 2-HG levels leads to the reactivation of epigenetic regulatory enzymes, including TET family dioxygenases, which contributes to DNA demethylation and partial normalization of gene expression patterns. These effects may enhance tumor differentiation, decrease malignant potential, and improve sensitivity to other therapeutic modalities [117,118,119,120,121,122,123,124].

Compared with TMZ—whose efficacy is strongly influenced by MGMT promoter methylation and which causes frequent hematologic toxicity—ivosidenib demonstrates a more favorable safety profile, with a relatively low incidence of severe adverse events such as neutropenia or thrombocytopenia. This makes it a promising option for patients who are ineligible for alkylating chemotherapy or for use in combination strategies in IDH-mutant diffuse gliomas [130,131,132,133,134,135,136,137,138].

Preclinical and early clinical evidence also supports the potential utility of ivosidenib both as monotherapy and in combination with radiotherapy or targeted agents, offering new therapeutic possibilities for IDH-mutant diffuse gliomas resistant to conventional treatment. Furthermore, emerging translational data suggest that ivosidenib-mediated modulation of the epigenetic landscape may enhance tumor immunogenicity, partly through increased expression of surface antigens, thereby improving responsiveness to immunotherapy. Current research is exploring combinations of ivosidenib with immune checkpoint inhibitors (PD-1/PD-L1) as a potential integrative treatment approach in IDH-mutant gliomas [130,131,132,133,134,135,136,137,138].

#### 2.4.3. Current State of Research

Ivosidenib was originally developed by Agios Pharmaceuticals as a first-in-class, small-molecule inhibitor selectively targeting mutant IDH1. Early structure-based drug design and biochemical studies demonstrated its high selectivity for mutant IDH1 over the wild-type enzyme, with effective suppression of 2-HG production in preclinical models [139,140,141,142]. The compound was subsequently evaluated in a series of preclinical studies, which confirmed its ability to reverse epigenetic abnormalities and promote differentiation in IDH1-mutant tumor cells [143,144].

Based on these findings, ivosidenib entered clinical development and was approved by the U.S. Food and Drug Administration in 2018 for the treatment of relapsed or refractory acute myeloid leukemia with IDH1 mutations, providing important proof-of-concept for its clinical safety and biological activity [145]. These results supported the subsequent exploration of ivosidenib in IDH1-mutant diffuse gliomas, including tumors historically described as secondary glioblastomas [145,146,147,148].

Clinical research on ivosidenib in IDH-mutant diffuse gliomas (currently classified as astrocytoma, IDH-mutant, including grade 4 in the WHO 2021 classification) remains in early development (Phase I/II), with trials focusing primarily on patients with confirmed IDH1 mutations. These tumors were historically described in part as “secondary glioblastoma”; however, under the WHO 2021 framework they represent a distinct entity and should not be classified as glioblastoma. This makes them a rational target group for precision therapies directed at metabolic and epigenetic vulnerabilities [137].

Preclinical studies in mouse models have shown that treatment with ivosidenib leads to a substantial reduction in tumor burden, accompanied by decreased levels of the oncometabolite 2-HG and partial restoration of gene expression programs associated with cellular differentiation [138]. These effects support the drug’s epigenetic mechanism of action and its ability to reverse IDH1-driven metabolic dysregulation.

Early Phase I clinical trials in humans have demonstrated that a proportion of patients achieve durable stable disease (SD), with progression delayed for several months beyond what is commonly observed in standard therapy for IDH-mutant diffuse gliomas [137]. While comprehensive data on overall survival (OS) remain limited, ongoing Phase II and III trials aim to clarify the long-term therapeutic value of ivosidenib and its influence on clinical outcomes in larger patient cohorts [137].

Promising research directions include combination strategies, such as pairing ivosidenib with angiogenesis inhibitors like bevacizumab, with the goal of improving drug delivery through normalization of pathological tumor vasculature [137]. In parallel, preclinical studies are examining the integration of ivosidenib with TTFields, which have demonstrated synergistic effects by disrupting mitotic processes and potentially enhancing treatment sensitivity [149].

Additionally, translational studies suggest that ivosidenib-induced modulation of the epigenetic landscape may increase tumor immunogenicity, including upregulation of surface antigens and improved responsiveness to immune checkpoint inhibitors [150]. These findings support ongoing efforts to evaluate ivosidenib in combination with PD-1/PD-L1 inhibitors as part of emerging multimodal therapeutic strategies in IDH-mutant gliomas.

#### 2.4.4. Clinical Perspectives

The introduction of ivosidenib into treatment strategies for IDH-mutant diffuse gliomas (including astrocytoma, IDH-mutant, grade 4) may represent a significant advance in the management of tumors driven by IDH1 mutations. Importantly, these tumors are distinct from glioblastoma, IDH-wildtype, under the WHO 2021 classification. Unlike conventional chemotherapeutic agents, ivosidenib exerts both metabolic effects—through inhibition of 2-HG production—and epigenetic effects by restoring more physiological patterns of gene-expression regulation. This aligns with the concept of precision medicine, offering a therapeutic approach tailored to the tumor’s molecular characteristics [135,136,138,139,140,141,142,143,144,145,146,147,148,149,150,151,152,153].

Successful clinical implementation of ivosidenib, however, will require further studies addressing dose optimization (currently evaluated in the range of 250–500 mg daily) and understanding its pharmacokinetic interactions with other agents commonly used in glioblastoma patients, including glucocorticosteroids and antiepileptic drugs, which may influence drug metabolism. Another essential direction involves identifying predictive biomarkers, such as circulating or intratumoral 2-HG levels, which could enable precise selection of patients most likely to benefit from therapy [135,136,149,150,151].

A promising area of investigation is the neoadjuvant use of ivosidenib, in which administration before surgical resection may decrease tumor burden and enhance operative conditions an approach currently under evaluation in Phase II clinical trials. Moreover, the potential applicability of ivosidenib extends beyond, glioblastoma as IDH1 mutations are also characteristic of lower-grade diffuse gliomas, suggesting that this targeted therapy may become relevant across a broader spectrum of IDH-mutant gliomas [151].

#### 2.4.5. Challenges and Limitations of Ivosidenib Use in Glioma Treatment

##### Bioavailability and the Blood–Brain Barrier

One of the main limitations of ivosidenib therapy’s effectiveness in brain tumors is its limited ability to cross the BBB, which results in relatively low drug exposure in tumor tissue compared to plasma and may hinder full inhibition of the oncometabolite 2-hydroxyglutarate production. Preclinical studies have shown that this inhibitor demonstrates only moderate brain penetration in animals, suggesting that in an intact BBB, drug availability to the central nervous system may be limited. In clinical trials of the new inhibitor Vorasidenib, designed for improved CNS penetration, the authors indicate that the relatively low brain exposure of ivosidenib was one of the reasons for vorasidenib’s development—which indirectly confirms ivosidenib’s limitations in the context of brain tumors. Additionally, the heterogeneous structure of brain tumors—with areas of hypoxia, necrosis, irregular vascularization, and disrupted vascular microarchitecture—may further impede drug diffusion within the tumor, even if some of the drug crosses the BBB. Therefore, alternative strategies for improving the delivery of IDH inhibitors to the CNS are increasingly being described in the literature. These include nanosystems (liposomes, nanoparticles), techniques that bypass or modulate the BBB, such as nasal drug delivery (nose-to-brain), or direct administration to the tumor parenchyma via convection-enhanced delivery (CED). Such approaches aim to increase drug concentration in the tumor while limiting systemic exposure—which theoretically could improve treatment efficacy while reducing toxicity [152,153,154].

##### Tumor Resistance and Heterogeneity

The development of resistance to ivosidenib represents a major clinical challenge. Multiple mechanisms have been proposed to contribute to therapeutic failure, including tumor heterogeneity and the presence of glioma stem cells, which are characterized by their capacity for self-renewal, survival under therapeutic stress, and tumor repopulation after treatment. GSCs have been widely reported to exhibit broad resistance to conventional anticancer therapies, which has been attributed to features such as metabolic adaptability and enhanced DNA damage response mechanisms. Although these properties may theoretically influence the durability of responses to targeted therapies, including IDH inhibitors, direct evidence linking GSC-specific metabolic plasticity or DNA repair capacity to resistance against IDH inhibition remains limited and requires further investigation.

Another well-documented mechanism involves secondary mutations within the *IDH1* gene or the emergence of alternative metabolic and signaling pathways that bypass IDH1 inhibition. Studies have demonstrated that activation of compensatory signaling networks—particularly the PI3K/AKT/mTOR and MAPK pathways—can reduce the therapeutic impact of ivosidenib by supporting tumor cell survival despite effective suppression of 2-HG production. Furthermore, the molecular heterogeneity of gliomas, including spatial variability in the distribution of IDH1-mutant and IDH1-wild-type tumor cell populations, complicates prediction of treatment response. Some tumor regions may be dominated by subpopulations that either lack IDH1 mutations or harbor alterations such as EGFRvIII, which drive tumor growth independently of IDH-dependent metabolic reprogramming and are therefore inherently insensitive to IDH inhibition. In light of these observations, the development of prognostic and predictive biomarkers including real-time next-generation sequencing approaches capable of detecting emerging resistant clones or secondary mutations—is considered essential for optimizing patient selection and monitoring treatment response. Preclinical studies also suggest that combining ivosidenib with inhibitors targeting compensatory pathways, such as PI3K/AKT, may enhance therapeutic efficacy by preventing or reducing the development of resistance [155,156,157,158].

##### Toxicity and Adverse Events

Although ivosidenib is considered a drug with relatively low systemic toxicity, especially when compared to TMZ, its long-term use may still be associated with adverse events that can influence treatment tolerability and overall therapeutic effectiveness. The most frequently reported symptoms include general fatigue and gastrointestinal discomfort such as nausea, which are typically mild to moderate in severity and manageable with supportive care. A less common but clinically important complication is differentiation syndrome, a reaction characterized by rapid maturation of tumor cells accompanied by fever, respiratory symptoms such as pulmonary edema, and systemic inflammation. This syndrome is associated with cytokine release particularly IL-6 and requires prompt recognition and immediate medical intervention due to the risk of rapid clinical deterioration. Another important issue involves pharmacokinetic interactions. Co-administration of drugs such as dexamethasone may alter the metabolism of ivosidenib through induction of CYP3A4 enzymes, potentially reducing therapeutic drug exposure. This necessitates careful clinical monitoring, evaluation of co-medications, and consideration of dose adjustments when clinically indicated. Strategies to reduce treatment-related toxicity include early recognition and prophylactic management of differentiation syndrome for example, the use of corticosteroids in suspected cases as well as regular assessment of hematologic parameters and liver function throughout treatment. Close pharmacovigilance and ongoing monitoring are essential to maintain patient safety and ensure optimal therapeutic benefit [159,160,161].

##### Implementation Limitations and Clinical Availability

Implementation of ivosidenib in clinical practice faces economic and regulatory challenges. As a targeted drug with a complex manufacturing process, ivosidenib remains expensive and limited in availability in countries with lower healthcare funding levels, which exacerbates therapeutic inequalities. Additionally, the lack of standardized diagnostic protocols enabling the rapid detection of IDH1 mutations hinders appropriate patient selection and reduces treatment effectiveness. Regulatory approval by agencies such as the FDA and EMA requires results from randomized phase III trials confirming efficacy and safety in large populations, which currently delays the drug’s integration into standard glioblastoma treatment [162,163].

##### Strategies to Overcome Limitations

Numerous translational and technological strategies are being developed to overcome these barriers. The use of magnetic resonance spectroscopy (MRS) enables non-invasive, real-time monitoring of 2-HG levels with high diagnostic accuracy, supporting personalized dose optimization and assessment of therapeutic response. Preclinical studies suggest that combining ivosidenib with inhibitors of key signaling cascades, such as PI3K or MEK, may have synergistic effects by enhancing pathway blockade and reducing tumor cell proliferation compared to monotherapy. In efforts to improve bioavailability, nanoparticle-based formulations and biodegradable implants capable of releasing the drug directly into the tumor environment have demonstrated promising outcomes by enhancing local drug accumulation within the target tissue. Further progress in this field is supported by international research collaborations, including initiatives promoted by organizations such as the European Society for Medical Oncology (ESMO), which help accelerate clinical data collection and reduce translational barriers [164,165,166].

## 3. Conclusions

The analysis of glioblastoma conducted in this paper allows for a comprehensive discussion of key aspects of this disease—from its pathophysiology to potential therapeutic strategies. Particular attention is paid to peritumoral edema, a significant clinical component of glioblastoma resulting from blood–brain barrier disruption and inflammatory processes. This phenomenon directly impacts the severity of neurological symptoms, prognosis, and therapeutic efficacy, significantly reducing patients’ quality of life.

Analysis of histopathological features highlighted the structural complexity of the tumor, including the presence of pleomorphic tumor cells, areas of necrosis, and microangiopathy, which are crucial for diagnosis and treatment planning integrated with molecular data. The paper further discusses current pharmacotherapy options, including, among others, chemotherapy. TMZ, dexamethasone, and bevacizumab, while beneficial in symptom control and prolonging time to progression, still face significant limitations related to tumor resistance, toxicity, and poor penetration across the blood–brain barrier.

The introduction of ivosidenib as a molecularly targeted drug candidate has opened up a new therapeutic perspective, especially in the treatment of secondary glioblastoma with an IDH1 mutation. Its mechanism, based on the inhibition of 2-hydroxyglutarate production and the restoration of epigenetic balance, represents a manifestation of precision medicine, distinguishing it from classical cytotoxic therapies. Phase I trials confirmed a 90% reduction in 2-HG levels and disease stabilization in 30% of patients, indicating significant potential for this therapy, although full confirmation of its efficacy requires results from phase III trials. Identified clinical challenges, such as limited bioavailability in the brain, tumor heterogeneity, resistance development, and toxicological and economic aspects, currently constitute major barriers to ivosidenib implementation. At the same time, emerging innovative strategies, including the use of nanoparticles, combinations with PI3K/MEK inhibitors, and modern imaging techniques, offer real opportunities to overcome these challenges. Current knowledge indicates that effective glioblastoma treatment requires an integrated and personalized approach, combining surgery, radiotherapy, and pharmacotherapy with molecular diagnostics and precise patient stratification. Challenges such as the blood–brain barrier, cellular resistance, and treatment costs remain key limitations. However, advances in drug delivery technologies, biomarker identification, and international research collaborations offer hope for improved quality of life and extended survival for glioblastoma patients.

## Figures and Tables

**Table 1:** Global incidence and mortality rates of glioblastoma in selected regions of the world [40,41,42,43,44,45,46,47].

Geographic Region	Incidence (per 100,000 People/Year)	Mortality Rate (per 100,000 People/Year)	Median Age of Onset (Years)	Clinical Notes
North America	3.2–3.5	2.8–3.0	63–66	Highest detection rate; well-developed diagnostics
Western Europe	3.0–4.0	2.5–3.0	60–65	High incidence in the elderly population
East Asia	0.8–1.2	0.5–0.9	58–62	Lower rates; possible genetic factors
South America	1.2–1.8	1.0–1.6	59–63	Limited access to diagnostic imaging
Sub-Saharan Africa	1.0–1.6	0.8–1.4	55–60	Likely underestimation of cases

**Table 2:** Selected pharmacological agents used in the management of glioblastoma and a qualitative summary of their clinical outcomes. The table summarizes the general clinical benefits and limitations of commonly used and investigational therapies in glioblastoma, based on findings discussed in the main text [73,74,75,76,77,78,79,80,81,82,83,84,85,86,87,88,89,90,91,92,93,94,95,96,97,98,99,100,101,102].

Drug	Class	Mechanism of Action	Clinical Application	Clinical Outcome	Common Side Effects
Temozolomide	Chemotherapeutic agent	DNA alkylation and induction of apoptosis	Standard adjuvant and concomitant therapy	Modest improvement in survival in newly diagnosed glioblastoma	Nausea, thrombocytopenia
Dexamethasone	Glucocorticosteroid	Reduction in vascular permeability and inflammation	Management of peritumoral edema	Symptomatic benefit without direct antitumor effect	Muscle weakness, hyperglycemia
Levetiracetam	Anticonvulsant	Stabilization of ion channels	Seizure control	Improvement of neurological symptoms and quality of life	Drowsiness, fatigue
Bevacizumab	Anti—VEGF antibody	Inhibition of tumor angiogenesis	Recurrent glioblastoma	Improvement in progression-free disease control without consistent survival benefit	Hypertension, bleeding

## Data Availability

The original contributions presented in this study are included in the article. Further inquiries can be directed to the corresponding authors.
